# Medical education departments: a study of four medical schools in Sub-Saharan Africa

**DOI:** 10.1186/s12909-015-0398-y

**Published:** 2015-07-01

**Authors:** Elsie Kiguli-Malwadde, Zohray M. Talib, Hannah Wohltjen, Susan C. Connors, Jonathan Gandari, Sekelani S. Banda, Lauren A. Maggio, Susan C. van Schalkwyk

**Affiliations:** 1 The MEPI Coordinating Centre at the African Centre for Global Health and Social Transformation, Kampala, Uganda; 2 MEPI Coordinating Center in the Department of Health Policy at the George Washington University, Washington, DC USA; 3 School of Education and Human Development, University of Colorado, Denver, USA; 4 College of Health Sciences, University of Health Sciences, Harare, Zimbabwe; 5 Department of Medical Education Development, University of Zambia, Lusaka, Zambia; 6 Lane Medical Library, Stanford University, Stanford, USA; 7 Centre for Health Professions Education, Faculty of Medicine and Health Sciences, Stellenbosch University, PO Box 241, Cape Town, 8000 South Africa

## Abstract

**Background:**

Many African countries are investing in medical education to address significant health care workforce shortages and ultimately improve health care. Increasingly, training institutions are establishing medical education departments as part of this investment. This article describes the status of four such departments at sub-Saharan African medical schools supported by the Medical Education Partnership Initiative (MEPI). This article will provide information about the role of these institutional structures in fostering the development of medical education within the African context and highlight factors that enable or constrain their establishment and sustainability.

**Methods:**

In-depth interviews were conducted with the heads or directors of the four medical education departments using a structured interview protocol developed by the study group. An inductive approach to analysis of the interview transcripts was adopted as the texts were subjected to thematic content analysis.

**Results:**

Medical education departments, also known as units or centers, were established for a range of reasons including: to support curriculum review, to provide faculty development in Health Professions Education, and to improve scholarship in learning and teaching. The reporting structures of these departments differ in terms of composition and staff numbers. Though the functions of departments do vary, all focus on improving the quality of health professions education. External and internal funding, where available, as well as educational innovations were key enablers for these departments. Challenges included establishing and maintaining the legitimacy of the department, staffing the departments with qualified individuals, and navigating dependence on external funding. All departments seek to expand the scope of their services by offering higher degrees in HPE, providing assistance to other universities in this domain, and developing and maintaining a medical education research agenda.

**Conclusions:**

The establishment of medical education departments in Sub-Saharan Africa is a strategy medical schools can employ to improve the quality of health professions education. The creation of communities of practice such as has been done by the MEPI project is a good way to expand the network of medical education departments in the region enabling the sharing of lessons learned across the continent.

## Background

Sub-Saharan Africa (SSA) suffers a disproportionate share of the world’s burden of disease while also experiencing a significant health care workforce shortage [1]. In addition, the dearth of medical schools in the region threatens efforts to scale up and improve the quality of medical education and health professions education (HPE) more broadly [2]. Yet, improving HPE is essential to the development of a robust workforce [3].

The demand for more physicians globally, coupled with the need for high quality education, has led to the proliferation of medical education departments across the world [4]. These departments (also called medical education research departments or centers) have many roles, including supporting medical education research, teaching, program evaluation, and facilitating the use of educational technologies [5], and may also impact at multiple levels (undergraduate, postgraduate, and continuing medical education) [4].

Medical education departments first appeared in Europe, but have since spread to programs in countries all over the world [6–12]. In general, there are few publications describing the role and development of these departments, and those available are often limited to descriptions of departments in non-African countries with one exception as described by Ofoegbu and Ozumba at the University of Enugu in Nigeria [13]. Given the intensifying demands on African medical schools to meet health workforce needs, more information is needed about such departments in the African context.

The history of medical education in Africa is, however, fairly recent [14]. Few African medical schools were established before 1960 and, while some were founded during the independence decades (1960–79), little growth occurred during the 1980s. Since then, the number of medical schools in Africa has increased significantly. By 2010, 166 medical schools existed in Africa with some reporting the presence of medical education departments [3, 14, 15]. Many African countries are investing in medical education as a key intervention to improve health care.

In 2010, the United States government launched the Medical Education Partnership Initiative (MEPI - http://www.mepinetwork.org/). This program provides financial support to 13 SSA medical schools over a five-year period to boost health worker education and strengthen national health systems [1]. The Initiative’s main objectives are to develop capacity in medical schools, retain the workforce, and build research capacity. MEPI schools were required to develop and implement medical education interventions and evaluate their effectiveness. Nine of the 13 MEPI schools chose to establish or strengthen medical education departments to support these efforts. Over the course of the Initiative, it has become apparent that sound and well-functioning medical education departments have the potential to provide critical support to institutions and health professionals in their medical education endeavors, as well as enhance medical education research that can further support MEPI and related initiatives. This paper provides an overview of four medical education departments. It also describes specific enablers and constraints that accompany both the establishment and sustainability of such departments with a view to informing medical schools in Africa and other under-resourced contexts, that wish to establish or strengthen a department of medical education. Table 1 provides the background of the four schools in this study.

**Table 1:** Overview of the four medical education departments in this study

School/college/Faculty name	1	2	3	4
	School of Medicine, University of Zambia, Zambia	Faculty of Medicine and Health Sciences, Stellenbosch University, South Africa	College of Medicine, University of Ibadan, Nigeria	College of Health Sciences, University of Zimbabwe, Zimbabwe
University established	1966	1866	1948	1952
First medical student intake	1968	1956	1954	1963
HPE programs offered	Nursing, Pharmacy, Biomedical Sciences, Environmental Health & Physiotherapy.	Physiotherapy, Occupational Therapy, Dietetics, Speech-Language & Hearing Therapy.	Dentistry, Nursing, Physiotherapy & Human Nutrition and Dietetics.	Nursing, Dentistry, Pharmacy, Public Health, Radiography, Medical Laboratory Science & Rehabilitation Science.
ME or HPE department established	2000	2006	2012	2014
Reason for establishing ME or HPE department	Following the merger of two complimentary teaching support units	Location away from main campus which had a functional unit. Growing need to support teaching and learning	To induct and train new staff	To support ongoing curricular reforms
Person in charge	Head	Director	Head	Director
Reporting structure	Dean	Dean	Provost	Dean
Staff structure (including directors)	7 academics	6 academics	4 academics	3 academics
	1 administrator	4 administrators	2 administrators	3 administrators

## Methods

This article explores the current status of the four medical education departments and offers a historical perspective of their development. The case examples were selected based on the schools’ active participation within the MEPI Medical Education Research Technical Working Group and included (in chronological order of the establishment of their medical education departments):

1. School of Medicine, University of Zambia, Zambia (UNZA)
2. Faculty of Medicine and Health Sciences, Stellenbosch University, South Africa (SU)
3. College of Medicine, University of Ibadan, Nigeria (UI)
4. College of Health Sciences, University of Zimbabwe, Zimbabwe (UZCHS)

When this study was conducted in 2012, these four institutions had departments that were either already established or were in development and their directors could therefore offer both historical and current perspectives on their departments.

Initially, a questionnaire was designed to collect data from the four sites, however, the development of appropriate questions became too complex to adequately capture the specific context of each institution. Therefore, the authors, all allied to the MEPI project, chose to conduct a qualitative study using structured interviews to better understand the nuances and examples peculiar to each site. The interview protocol was developed by the author team, and included a structured design to provide consistency as different members of the team conducted the interviews across the sites (Appendix A). The protocol reflected the evolution from questionnaire to interview format, and although there were relatively few open-ended questions posed, the interviewers were encouraged to probe to elicit further details. The transcriptions of the interviews provided much richer responses than could have been obtained with using a questionnaire-type format. The principal investigator on each school’s MEPI grant was asked to identify the appropriate interviewees. All interviewees (*n* = 4) held positions as the directors or heads of the medical education departments at the selected sites. Ethical approval was obtained from the participating institutions as required (for example: SU: N13/07/113). Interviewees were asked to complete a consent form and informed that their participation was voluntary.

All interviews were recorded and transcribed, and varied in length from one hour to 90 min. The data were subjected to thematic content analysis [16] by one member of the study (SCvS) who developed a code list using *Atlas ti* while a second (ZMT) developed a second code list through manual analysis. These two members compared their initial analyses, finalized the codes, and then completed the process of categorization. Team members who had conducted the interviews verified the outcome.

Although only four interviews were conducted, the authors believe that this study generates sufficient data to provide a picture of the status of medical education departments situated in medical schools in Africa. The intention was to not to evaluate, but to identify the enablers and constraints that are experienced in these contexts in the hope that this might provide insight for others on the continent.

## Results

Interviews conducted with lead faculty from the four medical education departments provide insight into the establishment and development, their mission as well as challenges they faced.

### Motivators for the establishment of medical education departments

The motivation to establish each of the four departments was quite different. At the University of Zambia, the Medical Education Department was created in 2000 (making it the oldest of the four studied) as an efficient solution to replace two complementary teaching departments (the Medical Illustration Department and the Teaching Development Department). The lead for the Department developed a concept paper, enlisted support from senior medical school leadership, and then secured external funding for advanced training in medical education. The Department was then formed and staffed with doctoral students interested in medical education. At Stellenbosch University, the motivation was different. The main University campus had a well-functioning teaching and learning unit, but the medical school was located on a different campus. The growing need for support in teaching and learning served as a catalyst for medical school faculty to start their own department. At the University of Zimbabwe, the establishment of the department was driven by a need to provide institutional support to ongoing curriculum reform. In Nigeria, the University of Ibadan first established their medical education department in 2012 to induct and train new faculty. In all cases, the director or head was the catalyst for the departments serving as a champion for education, and these individuals’ efforts were supported by the availability of funding and the prevailing momentum for innovation in medical education. Interviewees described the process as lengthy and complex, noting that establishment was often preceded by a period of negotiation and planning.

### Setting up a medical education department – leadership and staffing

The medical education departments at the four schools have different names (three called Departments, and one called a Center), often reflecting the position of the department in the institution [4, 15]. In all cases, a single person serves as a director or a coordinator for the departments. All departments are positioned within a medical school and ultimately report to a dean or head of the school. Staff numbers and appointments differ considerably across the departments (Table 1). The more established departments have a cadre of academic and non-academic staff, working either full or part-time. Across the institutions, the academic staff have a variety of academic backgrounds, including PhDs in HPE or Education, experience as clinician educators, and other educational qualifications. It is, however, important to note that often many of the staff members have been employed with external funding. Given the small staff complements, reporting structures are horizontal with academic staff often being responsible for a particular role. For example, at Stellenbosch, staff responsibilities include ‘student support,’ ‘faculty development,’ and ‘medical education research’.

### The mission and activities of medical education departments

All four schools use their departments to build capacity for their faculty in HPE, to foster scholarship in teaching and learning, and to support the process of curriculum review and renewal. Interviewees described the over-arching role of the department as enhancing student learning and improving faculty skills as medical educators. As one interviewee stated, “The mission of HPE is to promote professionalism and excellence in health professions education”. The departments are driven by a desire to promote social accountability (i.e. placing priority on the health needs of citizens and societies) and introduce innovative teaching strategies such as community-based education, problem-based learning, workplace-based learning and inter-professional education. To achieve these aims, the departments offered a wide range of activities (Table 2).

**Table 2:** Summary of the activities of the four medical education departments canvassed in this study

Function / activity	Departments			
	1	2	3	4
Build capacity for faculty in HPE	x	x	x	x
Support the development of scholarship in teaching and learning	x	x	x	x
Support curriculum development, renewal and implementation	x	x	x	x
Offer Postgraduate diplomas, Masters and Doctorate degrees in HPE	x	x	x	
Student academic support	x	x		x
Mentor faculty and provide educational advice	x	x		
Provide quality assurance at the institution and affiliated schools	x		x	
Consult on education matters for the region	x			
Coordinate research in medical education	x	x		
Manage the clinical skills laboratory	x	x		

### Challenges to establishing medical education departments

Interviewees reported that establishing a medical education department presented numerous challenges. One of the main issues identified was establishing and maintaining the legitimacy of the department within faculty and university leadership. As a discipline, medical education was reported to be philosophically different from other medical school disciplines. While most medical disciplines draw on a biomedical science model and predominantly engage with research in a positivist paradigm, education draws its philosophical roots from psychology and sociology. Medical education, therefore, requires working across disciplines both within and outside health. One respondent, in describing this tension and also emphasized the importance of engaging in activities such as international collaboration to enhance faculty and leadership perceptions of the department:

The biggest challenge was that at inception the concept of medical education was new to most members of staff who had a good 20–25 years [experience] and had not heard about the medical education services the department is providing. As a result, [there was] suspicion and resistance and uncertainty to where it was going but with more exposure to international communities and training in other universities, the acceptance uptake has been very high because they have seen that this is happening elsewhere.

In addition to establishing the credibility of the department, interviewees described a number of other challenges in running their departments including transient funding sources, the need to manage faculty expectations, and setting realistic goals while working towards an ambitious vision. Funding streams tend to be grant-based rather than institutional, placing the sustainability of the department at risk. Funding was required for physical infrastructure (such as office space, equipment, and telephones) as well as personnel. Department staff members are often appointed with ‘soft’ funding from grants and other outside sources. Despite the challenges of working with grant funding, there was acknowledgement of the value of external funding as it stimulated interest, activity, and support for the department both from within and outside of the institution. In this regard, the impact of MEPI was specifically noted as essential to the establishment and maintenance of the departments, especially at Ibadan and Zimbabwe. Some departments were able to expand their activities through MEPI funding, particularly in the areas of e-learning and medical education research. One interviewee described how the MEPI goals shaped the development of the medical education department:

We are going to work with the MEPI group so that we develop the department in line with the demands of MEPI which are: capacity-building, improving quality of medical education, retention and introduction of new methods of applying knowledge.

### Key enablers in establishing a medical education department

The challenges of establishing and maintaining medical education departments were mitigated through various strategies including engaging leadership and building key relationships (Table 3). The University of Zambia interviewee indicated that support from management was important because it facilitated “[jumping] through the different [bureaucratic] hoops”. Engaging faculty from different disciplines and from different departments was also seen as an enabler and critical to earning the sort of credibility that was described earlier. The interviewee from Stellenbosch reported that, because the work and research of the Center crossed disciplinary boundaries, the department had high visibility within the school. At the University of Zambia, the staff members from the medical education department collaborate with the library to support faculty in transferring lecture material to the e-learning platform. Exposing faculty to the discourse of medical education globally was seen as crucial to informing the work of the department and raising the standards of teaching and learning, specifically with regard to curriculum reform. Adequate funding for the department was also critically linked to recruiting and retaining academic staff members who can provide leadership for teaching faculty. Having a qualification in HPE or exposure to other advanced training in education was seen as necessary for both the leads and the staff of a medical education department. One interviewee explained, “I think it is an advantage, definitely an advantage to have someone with preferably a formal qualification in teaching”.

**Table 3:** Challenges and perceived enabling factors as reported by the four medical education departments in this study

Challenges	Enabling factors
Establishing and maintaining the legitimacy of the department within faculty and university leadership	Ensure academic status for the department
	Align with the school and institutional strategic foci
	Seek legitimacy for the department – acknowledged stature among faculty and within the school
Securing and retaining appropriate staff	Secure active and sustained support at senior level
	Hire staff who have expertise or qualifications in HPE
	Employ a qualified leader as champion
Sustaining the department in terms of funding	Build on the current regional call for medical education innovation
	Obtain funding from the institution and external grants
	Develop internal income-generating activities (e.g., postgraduate HPE programs which charge tuition)

### Measures of success

Interviewees pointed to a number of measures of success. Departments at the Universities of Stellenbosch, Ibadan, and Zimbabwe have evolved to become recognized academic departments and thus established entities in their institutions. Other successes include the growth in HPE postgraduate offerings, increased graduates from HPE programs, increased numbers of grants secured that can support the work in medical education departments, and the provision of support to other institutions on matters relating to medical education activities. Building up capacity to offer postgraduate degrees in HPE was cited as an important indicator of success.

The interviewee from the University of Zambia further explained the impact of the medical education department in terms of its potential to act as a change agent:

With regard to education innovation, we know that [medical education department] has been a prime player. For instance, the adoption of changing examination systems from the traditional long case and short case for our clinical courses was championed by the [department], for introduction of more objective and more varied assessment methods, including the objective strategy for clinical medical education. The drive to try and get the school do a self-evaluation against the World Federation of Medical Education standards was driven by [medical education department staff]. Also, the initiative to do a comprehensive curriculum review in the School of Medicine to begin to bring awareness of innovative educational methods and to change the curriculum from a totally traditional curriculum to start moving towards some innovative strategies was driven by [departmental staff]. So with regard to being a change agent, I think [medical education departments] plays a very key role.

### The future and sustainability of medical education departments

All interviewees had clear objectives for the future of their departments and reported seeing medical education as an area destined for expansion and increasing legitimacy. One interviewee at the University of Zambia viewed the work being done currently as a “pipeline issue” that would bear fruit in years to come. Developing and maintaining a medical education research agenda was a priority for all interviewees. Having a research output was considered critical to maintaining the credibility and significance of the department as well as achieving international visibility. The interviewee from Stellenbosch University felt it was important for the department to have a well-defined research focus and to identify faculty who would conduct research in this area. Collaboration around capacity-building initiatives, conferences, and more medical education journals for Sub-Saharan Africa, were also seen as desirable goals.

All interviewees articulated a need for developing future staff, particularly staff focused on medical education scholarship (e.g. PhD and Masters graduates). They also planned to continue capacity-building by offering workshops to provide advanced training in innovative teaching methods as well as mentoring for faculty members interested to pursue careers in medical education. The interviewee from the University of Ibadan reported plans to advocate for recognition of teaching excellence as a criterion for promotion, while the interviewee from Zimbabwe shared the hope of establishing a College of Health Professions Education to provide comprehensive postgraduate training. Finally, interviewees intended to ensure legitimacy of their respective departments by conducting rigorous evaluation of their activities.

## Discussion

The establishment of medical education departments that focus on enhancing the quality of physician training is a valuable initiative to support Africa’s health workforce needs [9, 10, 15]. MEPI has catalyzed widespread investment in medical education, and there has been a growth of medical education departments as schools seek to sustain and support these new educational activities. The availability of MEPI funding has stimulated both the creation and strengthening of medical schools’ infrastructure to support faculty development and scholarship in medical education and the role played by these medical education departments is evident from this research. At the four schools included in this study, the role of the departments are similar and are aligned with the MEPI goals of improving the quality and quantity of medical graduates and increasing capacity for research. They are also aligned with the Association for Medical Education in Europe’s charge to focus on research, faculty development in HPE, and support for learning and teaching [4, 17].

Although the four departments are at different stages of their development, they share common lessons learned in developing their respective departments. These include the importance of starting small, establishing relationships with key stakeholders in the institution, and developing credibility among colleagues and leadership. Ultimately, one of the indicators of success that will contribute to the value and sustainability of these departments is their ability to engage in medical education research. An aspiration of one of departments is to enhance the recognition of educational scholarship as a criterion for promotion. Such recognition has not been considered at many universities in the developing world in the past [18] and could serve as an important catalyst to further scholarly work in the field.

As medical schools grow in Africa, the need for global and regional engagement becomes more apparent. Therefore, fostering a community of practice is a key strategy in developing the field of medical education and supporting the necessary related institutional structures [19]. The MEPI network of schools has established a Technical Working Group on Medical Education Research, which provides a platform for SSA schools to network, share ideas, and engage in medical education initiatives. Activities that support and nurture this community of practice will be critical to facilitate the sharing of resources and lessons learned. Identifying best practices globally and in the region will enable their efficient and successful replication. Medical education staff and leaders are often pioneers in their institutions seeking to establish a new field of study. Joining regional or global communities with similar interests and challenges can fill the void while local communities and momentum are being developed.

All four directors recognized that capacity-building in HPE was an important component of their work and acknowledged the importance of having faculty leaders with advanced training in HPE. Similarly, experienced medical educators or clinicians played critical roles in building these departments, further validating the need for well-trained and motivated champions. These HPE experts serve an important advocacy role with the potential to create higher standards for medical education in the region, stimulate medical education research, and enhance the discipline’s standing. Fostering leaders in HPE who feel empowered as a result of being part of the community of practice provided by the medical education departments is critical in this context [19].

As has been reported in other developing countries [9], major challenges to the four African departments included in this study were limited infrastructure, funding, and faculty understanding. Leaders addressed these challenges by linking with senior management, establishing formal structures with their schools, and leveraging external funding to develop income-generating activities. These early findings from medical education departments in Africa suggest their sustainability will rely on the motivation of the medical education department leaders, support from university leadership, a sustained drive for curricular improvements, and creative funding sources.

The findings of this study should, however, be considered in light of the following limitations. All members of the study team were involved in the MEPI in various roles and, therefore, have an interest in supporting the success of medical education in Sub-Saharan Africa. Although this vested interest may have potentially biased the study team, all members were reflexive in their stance and approached the project as researchers focused on the research question. In addition, we recognize that our interviewees would have been predisposed to presenting their centers in a positive light. Nevertheless, we believe that the study provided an opportunity to reflect on the current status of medical education departments on the continent and that interviewees participated with this objective in mind. Lastly, institutions were selected based on their active participation within the MEPI Medical Education Research Technical Working Group. Future research might consider extending its reach to provide a broader spectrum of accounts.

## Conclusions

The establishment of medical education departments in SSA has taken root. Though relatively new in these four schools, the departments are already established in their respective institutions through the efforts of medical education champions and support from external funders and collaborators. Importantly, these departments have the potential to serve as catalysts to promote scholarship in the field, and to enhance the standard and relevance of medical education. Ultimately the aim is to strengthen the health work force to promote the quality of care for all.

## Appendix 1: Medical Education Department: Interview Guide

School:

A. Medical Education Department/Unit Background
  1. What is the Medical Education Department/Unit called at your institution?
  2. In what year was the Medical Education Department/Unit established?
  3. What are their primary goals of the Medical Education Department/ Unit or Medical Education Coordinator at your institution?
  4. Please describe the process of initiating the Medical Education Department/Unit at your institution.
    - What individual or individuals pushed the process?
    - Why was the development of a medical education department/unit deemed necessary?
    - What support was needed?
B. Staffing
  1. Is the Medical Education Department/Unit directed by a faculty member? If so, who is this individual?
    - Does this individual have advanced training in medical education? If so, please describe.
  2. How many full-time equivalent personnel are assigned to the Medical Education Department/Unit?
  3. How is the Medical Education Department/Unit staffed?
C. Funding
  1. What are the sources of funding for the Medical Education Department/ Unit?
D. Position within the Institution
  1. Within your institution’s structure, to whom does the Medical Education Department/Unit report?
  2. How would you describe the status of the Medical Education Department/Unit at your institution?
E. Activities
  1. What are the primary activities of the Medical Education Department/Unit at your institution?
    - Does the Medical Education Department/Unit have a relationship with the library at your institution? If so, please describe.
    - Does the Medical Education Department/Unit have a relationship with the unit that works on Instructional Technology (IT support) at your institution? If so, please describe.
    - To what extent is the Medical Education Department/Unit at your institution involved in medical education research?
    - To what extent does the Medical Education Department/Unit prepare students for a career as a medical educator? Please give an example.
    - To what extent does the Medical Education Department/Unit serve as a change agent within your institution (i.e. promotes organizations improvement for the purpose of achieving higher degrees of excellence)? Please give an example.
  2. To what extent is the Medical Education Department/Unit at your institution involved with grant programs funded by the Medical Education Partnership Initiative (MEPI)?
F. Overarching Evaluation
  1. Please describe any future plans for the Medical Education Department/unit at your institution.
  2. What factors have prevented or limited the implementation of a Medical Education Department/unit or Medical Education Coordinator at your institution?
  3. Have there been any specific successes stemming from the Medical Education Department/Unit? If so, please describe.
  4. Is there anything else you would like to share about the Medical Education Department/Unit at your institution?
